# Point-of-care testing and antibiotics prescribing in out-of-hours general practice: a register-based study in Denmark

**DOI:** 10.1186/s12875-024-02264-0

**Published:** 2024-01-23

**Authors:** Line Due Christensen, Claus Høstrup Vestergaard, Ellen Keizer, Bodil Hammer Bech, Flemming Bro, Morten Bondo Christensen, Linda Huibers

**Affiliations:** 1 Research Unit for General Practice, Bartholins Alle 2, 8000 Aarhus, Denmark; 2 Department of Public Health, Aarhus University, Bartholins Alle 2, 8000 Aarhus, Denmark

**Keywords:** Out-of-hours medical care, General practice, Denmark, Point-of-care testing, Anti-bacterial agents

## Abstract

**Background:**

Point-of-care testing may reduce diagnostic uncertainty in case of suspicion of bacterial infection, thereby contributing to prudent antibiotic prescribing. We aimed to study variations in the use of point-of-care tests (C-reactive protein test, rapid streptococcal antigen detection test, and urine dipstick) among general practitioners (GPs) and the potential association between point-of-care testing and antibiotic prescribing in out-of-hours general practice.

**Methods:**

We conducted a population-based observational register-based study, based on patient contacts with out-of-hours general practice in the Central Denmark Region in 2014–2017. The tendency of GPs to use point-of-care testing was calculated, and the association between the use of point-of-care testing and antibiotic prescribing was evaluated with the use of binomial regression.

**Results:**

Out-of-hours general practice conducted 794,220 clinic consultations from 2014 to 2017, of which 16.1% resulted in an antibiotic prescription. The GP variation in the use of point-of-care testing was largest for C-reactive protein tests, with an observed variation (p90/p10 ratio) of 3.0; this means that the GPs in the 90th percentile used C-reactive protein tests three times as often as the GPs in the 10th percentile. The observed variation was 2.1 for rapid streptococcal antigen detection tests and 1.9 for urine dipsticks. The GPs who tended to use more point-of-care tests prescribed significantly more antibiotics than the GPs who tended to use fewer point-of-care tests. The GPs in the upper quintile of the tendency to use C-reactive protein test prescribed 22% more antibiotics than the GPs in the lowest quintile (21% for rapid streptococcal antigen detection tests and 8% for urine dipsticks)*.* Up through the quintiles, this effect exhibited a positive linear dose–response correlation.

**Conclusion:**

The GPs varied in use of point-of-care testing. The GPs who tended to perform more point-of-care testing prescribed more antibiotics compared with the GPs who tended to perform fewer of these tests.

**Supplementary Information:**

The online version contains supplementary material available at 10.1186/s12875-024-02264-0.

## Background

Inappropriate antibiotic prescribing is linked to a risk of antibiotic resistance [1]. Point-of-care (POC) testing may reduce diagnostic uncertainty [2], thereby contributing to prudent antibiotic prescribing [3, 4]. Point-of-care tests are medical diagnostic tests performed at or near the site of care [5], such as measurement of C-reactive protein (CRP), enzyme immunoassay kits (e.g. rapid streptococcal antigen detection test (RADT)), and urine dipstick [6]. These tests can support general practitioners (GPs) in their clinical decision-making about the need for antibiotics [7–9]. In daytime general practice, the use of POC tests is widespread [6], but in out-of-hours (OOH) general practice access to and use of POC testing varies considerably between countries [10–14]. However, in recent years, the use of POC testing at OOH general practice has increased [8].

Antibiotic prescribing is influenced by a range of factors that are specific to OOH general practice, e.g., contacts more often concern vulnerable patients, foreign-language patients, and young children, with an overrepresentation of respiratory tract infections. Additionally, prescribers with a high level of antibiotic prescriptions were situated more often in deprived areas and rural settings [15]. Also, the workload is high, and there is limited access to patient records and diagnostics (incl. POC tests) [10, 16–18]. Moreover, GPs do not always feel confident enough to use the “wait and see” approach [18], making antibiotic prescriptions a safety net for the GPs [19]. Belgian GPs experienced a lower threshold of prescribing antibiotics in OOH general practice compared to office hours [20]. Some GPs of an English OOH home visiting service felt that POC testing could not add clinical value and that its use was associated with some practical challenges [13]. Yet, many studies showed that using a POC test can improve diagnostic accuracy and provide an objective validation for the GPs’ clinical assessment and decision-making [10, 21, 22], thereby reducing immediate antibiotic prescribing in both adults and children [10, 23–26].

More insight into GP variation in POC testing and the possible association between use of POC tests and antibiotic prescribing rates at OOH general practice can provide a knowledge base for future interventions that aim to facilitate prudent prescribing of antibiotics. Therefore, we aimed to study GP variation in the use of POC tests (i.e., CRP test, RADT, urine dipstick) and the association between use of POC testing and antibiotic prescribing at OOH general practice.

## Methods

### Design and population

We performed a population-based observational register-based study of all clinic consultations with OOH general practice of the Central Denmark Region from January 1, 2014 to December 31, 2017. We used routine data from the OOH general practice registration system and data from national registers. The study is reported according to the Strengthening and Reporting of Observational Studies in Epidemiology (STROBE) recommendations [27] (See Additional file 1).

### Setting

In Denmark, OOH general practice provides care to all regional citizens outside their regular GP’s office hours in four out of five regions. Primary care is tax-funded and thus free of charge at the point of service. Out-of-hours general practice is open on weekdays between 4 pm and 8 am, on weekends, and on holidays [28]. In the Central Denmark Region, OOH general practice has 11 locations for clinic consultations, often co-located with the emergency department. These 11 clinic locations have a similar overall organization, with some variation, for example regarding level of collaboration with nurses, access to POC tests, and size [29]. As general practice is responsible for providing care 24/7, GPs, and GP trainees, work in OOH general practice. Most GPs work also in daytime practice. Few physicians in OOH general practice have a different medical specialisation.

GPs are paid a fee-for-service [30], recording remuneration codes in the registration system of OOH general practice, including codes for conducting POC testing (e.g., CRP, RADT, and urine dipstick). When POC testing was introduced in Denmark, GPs had to bring tests to OOH general practice themselves. However, in recent years, first RADT and later CRP POC tests became available in the OOH clinic locations [31]. As such, Danish GPs have extensive experience using POC tests. Also, at all POC test machines in OOH clinic locations, user instructions are available.

### Data collection

We retrieved data directly from the OOH general practice registration system at contact level. For each clinic consultation, we received date and time of contact, patients’ age and sex, GPs’ age and sex, antibiotic prescription (Anatomical Therapeutic Chemical index, group J01: Antibacterial agents for systemic use) [32], and use of POC testing(i.e., CRP test, RADT, urine dipstick). The Civil Registration System, the Danish Education Register, and the Danish Income Statistics Register provided data on patient characteristics (i.e., ethnicity, marital status, urbanisation, income, and highest completed educational level). The National Health Insurance Service Register provided information on contacts with the daytime regular GP and the National Patient Register on diagnoses to calculate the number of Charlson comorbidities [33]. The Register of Authorised Health Personnel provided GPs’ years of registration and specialization.

We defined five variables, using data from OOH general practice. As a proxy of GP’s familiarity with working in OOH, we computed number of ‘OOH shifts in the past 180 days’. As a proxy for GP’s activity level at time of contact, we calculated ‘patients seen in the past hour’. These variables could not be calculated if the contact took place within the first 180 days of the study period (‘familiarity’) or during the first hour of the shift (‘activity’). We also constructed ‘patient GP and/or OOH contacts in the past 12 months’ as a proxy for the patients’ overall utilization of general practice. Finally, we defined ‘time to next in-hours period’, being the amount of time until daytime GP opening hours, and ‘regional patient load, past hour’, being the overall workload at the entire regional OOH general practice at the time of the contact.

### Analyses

Only clinic consultations at OOH general practice were included. We excluded GPs who had on average less than two shifts per year to avoid outliers. If necessary, a missing category was introduced for each covariate.

We calculated the tendency of GPs to use POC testing (PUT), for each GP and each type of POC test, by constructing a binomial regression model with POC test use as the outcome while adjusting for patient- and contact characteristics. This model allowed us to predict the expected number of used POC tests for each GP. Next, we defined PUT as the ratio of the actual (observed) number of used POC tests to this expected number of POC tests. A PUT > 1 means that the GP was more likely to use POC testing than the average GP and a PUT < 1 means that the GP was less likely to use POC testing than the average GP, adjusted for case-mix. To analyse whether GP characteristics were associated with the use of POC testing, we conducted a mutually adjusted analysis to estimate the relative PUT for different GP characteristics. Characteristics included in the model were: sex, age, years of experience as GP, primary care specialist, number of OOH shifts in past 180 days, and number of patients seen in past hour. As such, we will for example get an estimate male tendency to use POC testing compared to tendency for females.

Finally, we performed binomial regression to investigate the association between GP’s PUT and antibiotic prescription for each of the three POC tests. For this analysis, the PUT was calculated based on the last 100 OOH clinical contacts prior to the index contact. The results are presented as relative antibiotic prescribing rates with 95% confidence intervals (95% CI). All regression models included robust variance estimation on the GP level to account for repeated measurements. We performed all analyses in Stata 16 (StataCorp LP, College Station, TX, USA).

## Results

### Contact-, patient-, and GP characteristics

**Fig. 1:**
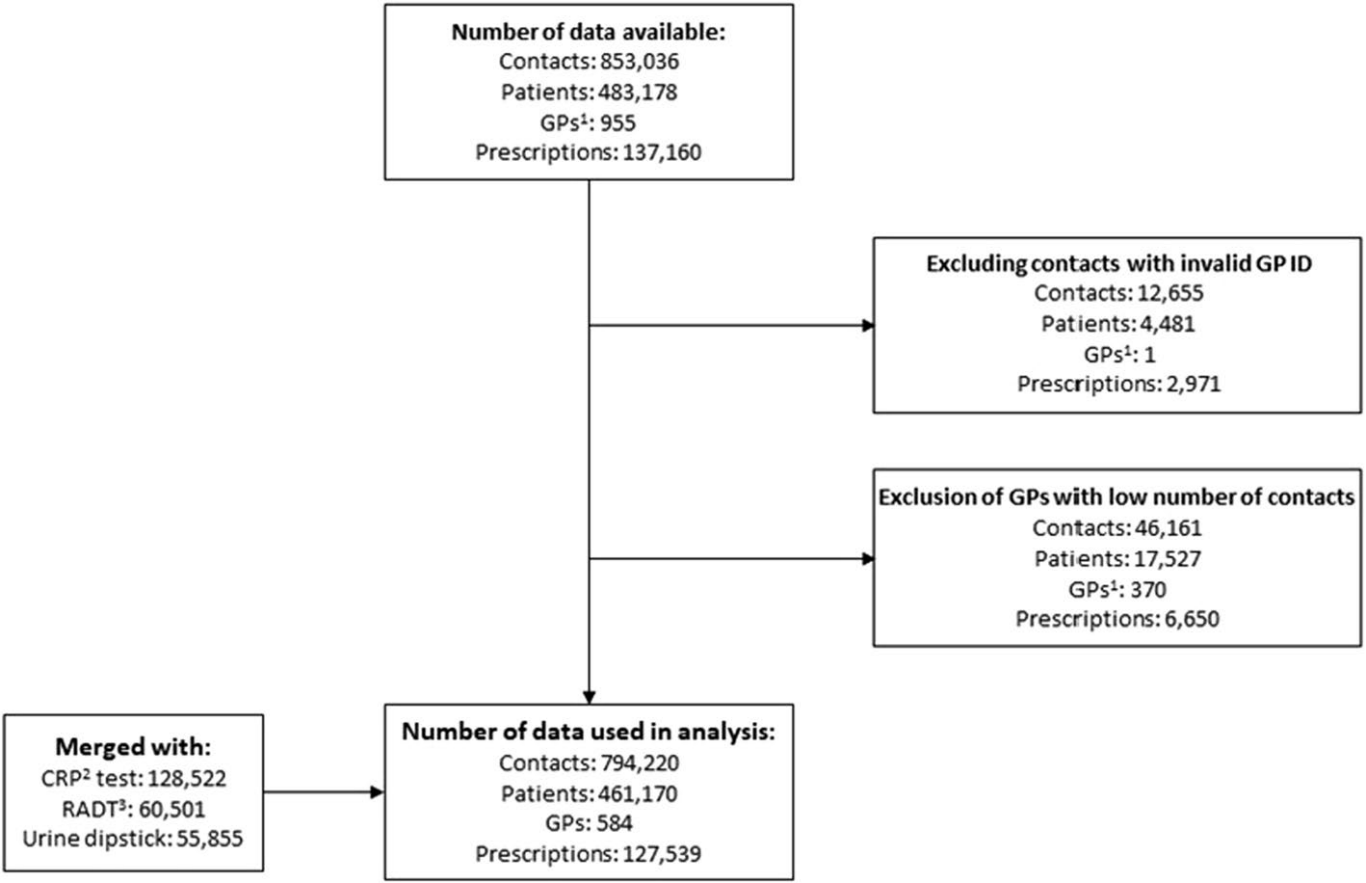
Flowchart of in- and exclusion of contacts ^1^GPs: general practitioners, ^2^CRP: C-reactive protein, ^3^RADT: rapid streptococcal antigen detection test

In the study period, OOH general practice in the Central Denmark Region had 794,220 clinic consultations, and 127,539 antibiotic prescriptions (Fig. 1). In 207,137 contacts one or more POC tests were performed: 128,522 CRP tests, 60,501 RADTs, and 55,855 urine dipsticks. Additional table A1 (Additional file 2) shows more details about contact- and patient characteristics for all clinic consultations, stratified by POC testing.

### GP POC test

**Fig. 2:**
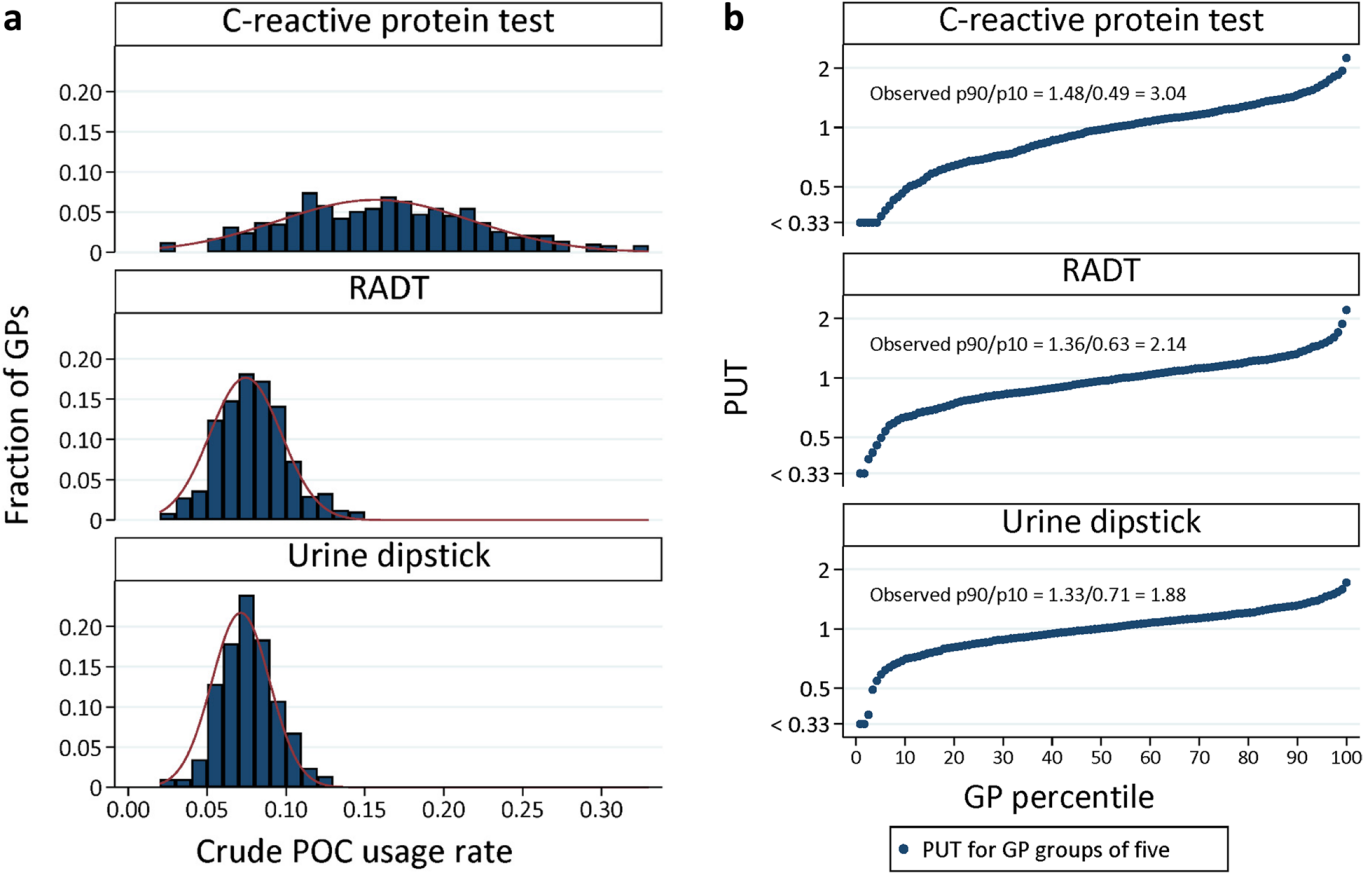
**a** Histogram for crude POC testing. **b** Tendency of GPs to use POC testing (PUT), measured as observed/expected for C-reactive protein, RADT, and urine dipstick respectively POC: point of care, PUT: Tendency of GPs to use POC testing, RADT: rapid streptococcal antigen detection test

Figure 2a presents the crude POC testing usage rate among GPs, whereas Fig. 2b shows the observed PUT for the three POC tests. The GP variation in use of POC testing was largest for CRP tests, with an observed variation (p90/p10 ratio) of 3.04, meaning that the 90th percentile GPs used CRP test three times as often as the 10th percentile GPs. For RADTs the observed variation was 2.14 and for urine dipstick 1.88.

### Use of POC testing and GP characteristics

**Fig. 3:**
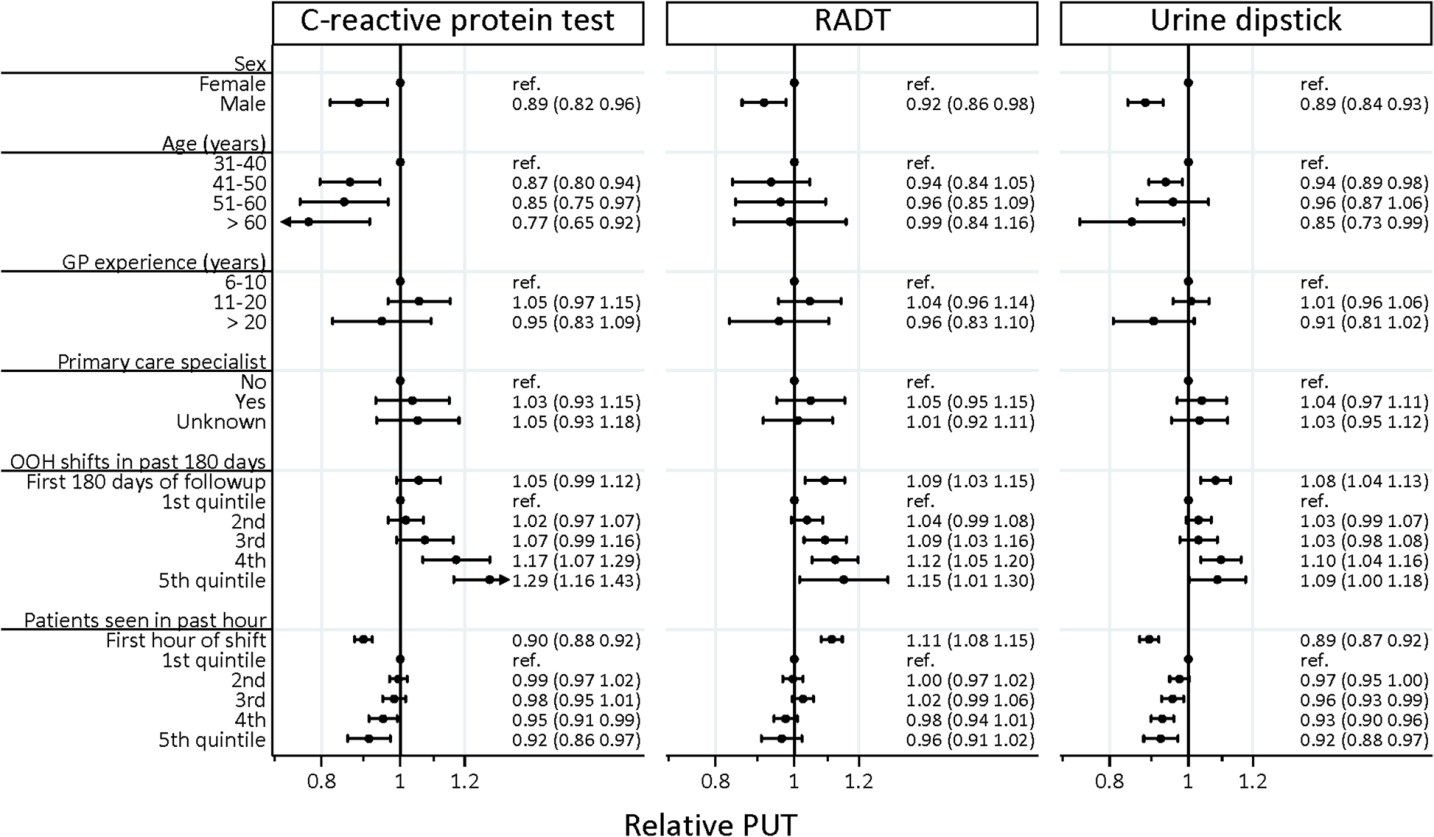
GPs’ tendency to use POC testing, for different GP characteristics, stratified by CRP test, RADT, and urine dipsticks. Mutually adjusted binomial regression (relative PUT, 95% confidence interval) PUT: Tendency of GPs to use POC testing, RADT: Rapid streptococcal antigen detection test

Figure 3 shows the association between GPs’ PUT and GP characteristics for CRP tests, RADTs, and urine sticks, respectively. Male GPs performed significantly fewer POC tests than female GPs (reference category), having a relative PUT of 0.89 [95% CI: 0.82;0.96] for CRP tests, of 0.92 [95% CI: 0.87;0.98] for RADTs, and 0.89 [95% CI: 0.84;0.93] for urine dipstick. Older GPs were significantly more likely to have lower levels of PUT for CRP tests (41–50 years: 0.87 [95% CI: 0.80;0.94], 51–60 years: 0.85 [95% CI: 0.75;0.97], > 60 years: 0.77 [95% CI: 0.65;0.92]) than younger GPs (reference 31–40 years). Further, GPs with few shifts were significantly more likely to have lower levels of PUT compared to GPs with more shifts. Additional figure A1 (Additional file 3) shows unadjusted results.

### Use of POC testing and relative antibiotic prescribing rate

**Fig. 4:**
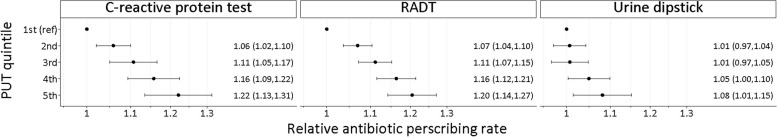
Relative antibiotic prescribing rate for different levels of GPs’ tendency to use POC testing (PUT), stratified by type of POC test. Fully adjusted binomial regression PUT: Tendency of GPs to use POC testing, RADT: Rapid streptococcal antigen detection test

General practitioners with higher levels of PUT tended to prescribe more antibiotics (Fig. 4). Compared to the GPs from the lowest quintile of PUT, GPs in the top PUT quintile had significantly higher relative antibiotics prescribing rate of 1.22 [95% CI: 1.13;1.31] for CRP, 1.20 [95% CI: 1.14;1.27] for RADT, and 1.08 [95% CI: 1.01;1.15] for urine dipstick. Up through the quintiles, this effect exhibited a positive linear correlation.

We performed stratified analyses on GPs’ sex and age for the association between GPs’ PUT and antibiotics prescribing rate. For sex, we did not find a modifying effect on the association, whereas a weak modification was present with increasing age for RADT (see Additional files 4 and 5).

## Discussion

### Statement of principal findings

General practitioners showed substantial variation in the use of CRP tests, RADTs, and urine dipsticks at OOH general practice. Male GPs performed fewer POC tests than female GPs. Additionally, GP age and number of shifts were also related to GPs’ tendency to use POC testing (PUT). Furthermore, we found a positive linear correlation between GPs’ PUT and their tendency to antibiotic prescribing.

### Strengths and limitations

The main strength of this study was the large dataset with unique data from OOH general practice on GP level, supplemented by data from the Danish national registries. With this data, we were able to study GP variation in the use of POC testing while adjusting for case-mix (i.e., patient- and contact characteristics), thereby limiting confounding. However, the study might still be subject to unobservable confounding, which might have influenced both the level of PUT and the number of antibiotics prescriptions. Possible confounding factors can occur at both organisational and personal levels. Also, GPs using an *immediate* strategy (GPs always take action on symptoms) in general, may also have an expected higher level of PUT and a higher antibiotic prescribing rate compared with GPs using a *wait-and-see* strategy in general.

Using Danish registers containing high-quality data ensured wide coverage and reduced the risk of selection bias. We did have to merge two independent datasets (i.e., one with prescriptions and one with contacts), resulting in a 3.8% loss of prescriptions due to a lack of matching contact. We only had data from one of five Danish regions, but regional variations are unlikely to influence the antibiotic prescribing patterns and the use of POC testing as the Danish population is fairly similar across the nation. Between countries, the level of variation may be related to organizational factors, including access to POC tests. Nevertheless, our results are likely to be useful outside Denmark and generalisable to other Western countries with similar healthcare systems. The precision of the prediction model might have improved, if we had data information on reasons for contacts, diagnoses, and POC testing results. Data at ICPC-coding [34] could fulfil this lack. However, ICPC-coding is not performed at Danish OOH general practice. Although this might have influenced the magnitude of our estimates slightly, it is unlikely to have systematically biased the direction. Finally, although we were able to study the association between POC testing and antibiotic prescribing, we lack knowledge of the decision-making process to test and prescribe.

### Findings in relation to other studies

As far as we know, no studies have explored GP variation in the use of POC testing at OOH general practices, but previous studies investigated variation in daytime practices, mostly on practice level, finding considerable variation [6, 12, 15, 35]. One Danish study found a more than fivefold inter-practice variation in the overall use of POC testing. Factors such as the patients’ age, the GPs’ age and sex, as well as geographical differences within Denmark were associated with the rate of POC testing [6]. This finding was in line with the up to threefold GP variation that we found. Furthermore, we also found that sex and age were associated with use of POC testing.

Our finding of the positive linear correlation between GPs’ use of POC testing and antibiotic prescribing at OOH general practice could have several explanations. More frequent use of POC testing could indicate use for cases with a low probability of bacterial infections, which reduces the validity of the diagnostic tests (i.e., lower positive predictive value, lower sensitivity). As a GP’s general attitude to pharmacotherapy seems important for antimicrobial chemotherapy [36], high-prescribers of antibiotics may also be high-prescribers of other medication, and possibly high-users of diagnostic tests [36]. Also, GPs may encounter more challenges with the interpretation of results particularly intermediate results, leading to misinterpretation and possibly non-optimal use of antibiotics [37]. The use of POC testing should be well-considered to avoid irrelevant use of POC testing and hence a waste of resources in a healthcare system [38, 39].

On the other hand, GPs could also experience barriers concerning use of POC testing, ranging from high workload, clinical utility, maintaining patient satisfaction, reimbursement, legislation, technical performance, connectivity, and training and maintenance [40, 41]. An interview study with GPs from six countries found that GPs worried that use of POC testing could lead to more patient-initiated consultations in the future [6, 40]. Hay [42] also referred to this, questioning whether POC testing should principally act as behaviour change tool by modifying patient expectations [43] and possible unintended consequences of POC testing such as medicalising self-limiting illnesses [44]. Some GPs of a British OOH home visiting service felt that POC testing did not add clinical value and that the use of POC testing incorporated some practical challenges [13]. However, a systematic review found that GPs believed that POC testing could increase diagnostic certainty and encourage decision-making and communication between GPs and patients [37]. A survey study from the Netherlands found comparable results, with Dutch GPs believing the proven effect on clinical management and the tests’ reliability to be among the most important aspects of POC testing [45]. A recent study found that getting experience with POC testing, and understanding where to use it clinically, supported further use [21]. However, GPs had concerns associated with test accuracy, GPs becoming too reliant on tests that could undermine clinical skills, and the limited usefulness of POC testing [37].

### Implications

We found GP variation in use of POC testing and a linear correlation between GPs’ level of POC testing and level of antibiotic prescribing, with higher levels of POC testing being correlated with more antibiotic prescriptions. More insight is needed before developing interventions that aim to facilitate prudent antibiotic prescribing. Qualitative research should focus on GPs’ decision-making process and their considerations for using POC testing and prescribing antibiotics or refraining from prescribing, as well as the GPs’ attributes and behaviours towards use of POC testing. Further studies should gain an in-depth knowledge of the rationale behind differences in the use of POC testing in OOH general practice among patients with focus on clinical conditions and sociodemographic factors. Furthermore, patients’ preference regarding antibiotic treatment and GPs’ factors such as experience, knowledge, empathy and preference, could be identified in such qualitative research, as studying GP characteristics via the crude register-based information in this study only explained a limited part of GP variation in use of POC testing. Identified modifiable GP and organisational factors could be operationalised and measured quantitatively to estimate their relative importance in explaining variation.

## Conclusion

We found considerable variation in the use of POC testing between GPs, implying room for improvement. Furthermore, GPs’ level of POC testing use was positively correlated with antibiotic prescribing, meaning that GPs who performed more POC testing also prescribed more antibiotics. Future research should focus on GPs’ decision-making process, GPs’ considerations for using POC testing and prescribing antibiotics or refraining from prescribing, and other relevant GP and organisational factors related to use of POC testing.

### Supplementary Information

**Additional file 1. **STROBE Statement—checklist of items that should be included in reports of observational studies.

**Additional file 2: Table A1. **Contact- and patient characteristics for all clinic consultations, stratified for use of POCTs.

**Additional file 3: Figure A1. **GPs’ tendency to use POC testing, for different GP characteristics, stratified by CRP test, RADT, and urine dipsticks. Unadjusted binomial regression (relative PUT and 95% confidence interval).

**Additional file 4: Figure A2. **Relative antibiotic prescribing rate for different levels of GP’s tendency to use POC testing (PUT), stratified by type of POC test and GP sex. Fully adjusted binomial regression (relative rate, 95% confidence interval).

**Additional file 5: Figure A3. **Relative antibiotic prescribing rate for different levels of GP’s tendency to use POC testing (PUT), stratified by type of POC test and GP age. Fully adjusted binomial regressions (relative rate, 95% confidence interval).

## Data Availability

The datasets generated and/or analysed in the current study are not publicly available as the data is stored at Statistic Denmark and is only accessible through a protected network connection in accordance with the Danish regulations of research.

## Abbreviations

CI: Confidence intervals
CRP: C-reactive Protein
GP: General practitioner
OOH: Out-of-hours
POC: Point-Of-Care
PUT: Tendency of GPs to use POC testing
RADT: Rapid streptococcal antigen detection test
