# Supplementary material for: Point-of-care testing and antibiotics prescribing in out-of-hours general practice: a register-based study in Denmark
Source: BMC Prim Care. 2024 Jan 23;25:31. doi: 10.1186/s12875-024-02264-0 (PMC10804570; doi:10.1186/s12875-024-02264-0)
Supplement: Supplementary file 2 — Additional file 2: Table A1. Contact- and patient characteristics for all clinic consultations, stratified for use of POCTs. [file 12875_2024_2264_MOESM2_ESM.docx]

**Additional file 2.**

**Table A1.** Contact- and patient characteristics for all clinic consultations, stratified for use of POCTs.

|  | | **All**^2^**^­­­^**  **No. (%)** | **POCT**^1^ | | | **No POCT**  **No. (%)** |
| --- | --- | --- | --- | --- | --- | --- |
|  |  |  | **CRP**^3^ **test**  **No. (%)** | **RADT**^4^  **No. (%)** | **Urine dipstick**  **No. (%)** |  |
|  | | 794,220 (100.0) | 128,522 (16.2) | 60,501 (7.6) | 55,855 (7.0) | 587,083 (73.9) |
| ***Contact characteristics*** | |  |  |  |  |  |
| **Year** | |  |  |  |  |  |
| 2014 | 200,497 (25.2) | 25,376 (19.7) | 15,633 (25.8) | 13,222 (23.7) | 153,694 (26.2) |  |
| 2015 | 203,326 (25.6) | 31,287 (24.3) | 16,271 (26.9) | 14,012 (25.1) | 151,059 (25.7) |  |
| 2016 | 198,227 (25.0) | 35,129 (27.3) | 14,573 (24.1) | 14,052 (25.2) | 144,455 (24.6) |  |
| 2017 | 192,170 (24.2) | 36,730 (28.6) | 14,024 (23.2) | 14,569 (26.1) | 137,875 (23.5) |  |
| **Month** | |  |  |  |  |  |
| January | 64,515 (8.1) | 11,148 (8.7) | 5,949 (9.8) | 4,608 (8.2) | 46,009 (7.8) |  |
| February | 63,150 (8.0) | 11,972 (9.3) | 6,340 (10.5) | 3,866 (6.9) | 44,136 (7.5) |  |
| March | 73,773 (9.3) | 12,967 (10.1) | 6,910 (11.4) | 4,594 (8.2) | 52,716 (9.0) |  |
| April | 74,010 (9.3) | 11,784 (9.2) | 6,664 (11.0) | 5,093 (9.1) | 54,002 (9.2) |  |
| May | 72,964 (9.2) | 10,413 (8.1) | 5,794 (9.6) | 4,858 (8.7) | 55,197 (9.4) |  |
| June | 67,842 (8.5) | 9,065 (7.1) | 4,481 (7.4) | 4,558 (8.2) | 52,611 (9.0) |  |
| July | 61,725 (7.8) | 9,177 (7.1) | 3,686 (6.1) | 4,653 (8.3) | 47,031 (8.0) |  |
| August | 65,569 (8.3) | 9,280 (7.2) | 3,288 (5.4) | 4,867 (8.7) | 51,021 (8.7) |  |
| September | 61,187 (7.7) | 9,163 (7.1) | 3,260 (5.4) | 4,404 (7.9) | 47,097 (8.0) |  |
| October | 62,099 (7.8) | 10,286 (8.0) | 3,894 (6.4) | 4,800 (8.6) | 46,194 (7.9) |  |
| November | 58,819 (7.4) | 9,944 (7.7) | 4,097 (6.8) | 4,568 (8.2) | 43,281 (7.4) |  |
| December | 68,567 (8.6) | 13,323 (10.4) | 6,138 (10.1) | 4,986 (8.9) | 47,788 (8.1) |  |
| **Time to next in-hours period** (hours) | |  |  |  |  |  |
| ≤16 | 350,484 (44.1) | 52,391 (40.8) | 21,328 (35.3) | 24,058 (43.1) | 268,657 (45.8) |  |
| 16-64 | 443,736 (55.9) | 76,131 (59.2) | 39,173 (64.7) | 31,797 (56.9) | 318,426 (54.2) |  |
| **Patient load regionally, past hour** (quintiles) | |  |  |  |  |  |
| First hour of shift | 65,316 (8.2) | 8,794 (6.8) | 4,818 (8.0) | 4,239 (7.6) | 50,181 (8.5) |  |
| 1^st^ | 150,601 (19.0) | 24,222 (18.8) | 10,893 (18.0) | 11,149 (20.0) | 111,646 (19.0) |  |
| 2^nd^ | 146,371 (18.4) | 23,395 (18.2) | 10,047 (16.6) | 10,605 (19.0) | 109,359 (18.6) |  |
| 3^rd^ | 148,032 (18.6) | 23,920 (18.6) | 10,653 (17.6) | 10,526 (18.8) | 110,109 (18.8) |  |
| 4^th^ | 140,562 (17.7) | 22,977 (17.9) | 11,039 (18.2) | 9,756 (17.5) | 103,401 (17.6) |  |
| 5^th^ | 143,338 (18.0) | 25,214 (19.6) | 13,051 (21.6) | 9,580 (17.2) | 102,387 (17.4) |  |
| ***Patient characteristics*** | |  |  |  |  |  |
| **Age in groups** (years) | |  |  |  |  |  |
| ≤3 | 123,626 (15.6) | 14,467 (11.3) | 9,874 (16.3) | 2,018 (3.6) | 99,968 (17.0) |  |
| 4-17 | 163,130 (20.5) | 18,917 (14.7) | 19,042 (31.5) | 9,074 (16.2) | 123,633 (21.1) |  |
| 18-39 | 253,280 (31.9) | 43,799 (34.1) | 22,379 (37.0) | 24,876 (44.5) | 178,342 (30.4) |  |
| 40-64 | 183,704 (23.1) | 36,246 (28.2) | 8,030 (13.3) | 12,898 (23.1) | 134,773 (23.0) |  |
| ≥65 | 70,480 (8.9) | 15,093 (11.7) | 1,176 (1.9) | 6,989 (12.5) | 50,367 (8.6) |  |
| **Sex** | |  |  |  |  |  |
| Female | 409,657 (51.6) | 72,574 (56.5) | 34,249 (56.6) | 40,844 (73.1) | 286,241 (48.8) |  |
| Male | 384,563 (48.4) | 55,948 (43.5) | 26,252 (43.4) | 15,011 (26.9) | 300,842 (51.2) |  |
| **Highest completed educational level** (years) | |  |  |  |  |  |
| <10 | 207,886 (26.2) | 37,705 (29.3) | 14,388 (23.8) | 17,964 (32.2) | 149,964 (25.5) |  |
| 10-15 | 230,679 (29.0) | 43,246 (33.6) | 14,689 (24.3) | 19,224 (34.4) | 165,755 (28.2) |  |
| >15 | 86,627 (10.9) | 16,307 (12.7) | 6,292 (10.4) | 8,106 (14.5) | 60,530 (10.3) |  |
| Children | 250,065 (31.5) | 27,884 (21.7) | 24,167 (39.9) | 8,606 (15.4) | 197,040 (33.6) |  |
| Unknown | 1,017 (0.1) | 144 (0.1) | 6 (0.0) | 92 (0.2) | 813 (0.1) |  |
| **Income**^5^ (deciles) | |  |  |  |  |  |
| 1st-3rd | 228,446 (28.8) | 36,798 (28.6) | 16,570 (27.4) | 18,187 (32.6) | 168,563 (28.7) |  |
| 4th-7th | 329,701 (41.5) | 54,604 (42.5) | 26,677 (44.1) | 22,506 (40.3) | 241,608 (41.2) |  |
| 8th-10th | 215,067 (27.1) | 35,250 (27.4) | 16,536 (27.3) | 14,639 (26.2) | 158,613 (27.0) |  |
| Unknown | 21,006 (2.6) | 1,870 (1.5) | 718 (1.2) | 523 (0.9) | 18,299 (3.1) |  |
| **Marital status** | |  |  |  |  |  |
| Married/cohabitating | 543,295 (68.4) | 88,884 (69.2) | 45,134 (74.6) | 37,179 (66.6) | 398,079 (67.8) |  |
| Single | 229,919 (28.9) | 37,768 (29.4) | 14,649 (24.2) | 18,153 (32.5) | 170,705 (29.1) |  |
| Unknown | 21,006 (2.6) | 1,870 (1.5) | 718 (1.2) | 523 (0.9) | 18,299 (3.1) |  |
| **Ethnicity** | |  |  |  |  |  |
| Native born | 678,818 (85.5) | 111,038 (86.4) | 52,518 (86.8) | 46,861 (83.9) | 500,641 (85.3) |  |
| 1^st^ generation immigrant | 57,638 (7.3) | 10,701 (8.3) | 3,696 (6.1) | 6,354 (11.4) | 40,529 (6.9) |  |
| 2^nd^ generation immigrant | 36,758 (4.6) | 4,913 (3.8) | 3,569 (5.9) | 2,117 (3.8) | 27,614 (4.7) |  |
| Unknown | 21,006 (2.6) | 1,870 (1.5) | 718 (1.2) | 523 (0.9) | 18,299 (3.1) |  |
| **Urbanisation** (inhabitants) | |  |  |  |  |  |
| >100,000 | 161,894 (20.4) | 23,100 (18.0) | 11,251 (18.6) | 13,083 (23.4) | 121,028 (20.6) |  |
| 20,000-100,000 | 219,290 (27.6) | 38,964 (30.3) | 18,484 (30.6) | 15,932 (28.5) | 157,591 (26.8) |  |
| 1,000-20,000 | 214,882 (27.1) | 35,054 (27.3) | 16,706 (27.6) | 14,846 (26.6) | 158,719 (27.0) |  |
| <1,000 | 174,718 (22.0) | 29,215 (22.7) | 13,247 (21.9) | 11,314 (20.3) | 129,494 (22.1) |  |
| Not placeable | 2,430 (0.3) | 319 (0.2) | 95 (0.2) | 157 (0.3) | 1,952 (0.3) |  |
| Unknown | 21,006 (2.6) | 1,870 (1.5) | 718 (1.2) | 523 (0.9) | 18,299 (3.1) |  |
| **Co-morbidity** | |  |  |  |  |  |
| No comorbidities | | 681,020 (85.7) | 104,347 (81.2) | 55,560 (91.8) | 46,894 (84.0) | 506,639 (86.3) |
| 1 | 87,498 (11.0) | 17,984 (14.0) | 4,402 (7.3) | 6,684 (12.0) | 62,556 (10.7) |  |
| 2 | 18,577 (2.3) | 4,405 (3.4) | 432 (0.7) | 1,666 (3.0) | 12,947 (2.2) |  |
| 3 | 5,072 (0.6) | 1,249 (1.0) | 86 (0.1) | 427 (0.8) | 3,543 (0.6) |  |
| ≥4 | 2,053 (0.3) | 537 (0.4) | 21 (0.0) | 184 (0.3) | 1,398 (0.2) |  |
| **Patient GP**^6^**/OOH**^7^ **contacts last 12 months** (quintiles) | |  |  |  |  |  |
| 1^st^ | 196,013 (24.7) | 24,429 (19.0) | 15,520 (25.7) | 8,703 (15.6) | 154,540 (26.3) |  |
| 2^nd^ | 170,531 (21.5) | 25,073 (19.5) | 14,887 (24.6) | 9,906 (17.7) | 128,241 (21.8) |  |
| 3^rd^ | 123,772 (15.6) | 20,240 (15.7) | 10,302 (17.0) | 8,556 (15.3) | 90,655 (15.4) |  |
| 4^th^ | 147,121 (18.5) | 26,796 (20.8) | 11,306 (18.7) | 12,187 (21.8) | 104,611 (17.8) |  |
| 5^th^ | 156,783 (19.7) | 31,984 (24.9) | 8,486 (14.0) | 16,503 (29.5) | 109,036 (18.6) |  |
| ***GP characteristics*** | |  |  |  |  |  |
| **Sex** | |  |  |  |  |  |
| Female | 305,467 (38.5) | 52,640 (41.0) | 24,070 (39.8) | 23,600 (42.3) | 221,123 (37.7) |  |
| Male | 488,753 (61.5) | 75,882 (59.0) | 36,431 (60.2) | 32,255 (57.7) | 365,960 (62.3) |  |
| **Age (years)** | |  |  |  |  |  |
| 31-40 | 154,484 (19.5) | 27,351 (21.3) | 12,215 (20.2) | 11,607 (20.8) | 111,684 (19.0) |  |
| 41-50 | 303,572 (38.2) | 52,133 (40.6) | 23,652 (39.1) | 22,353 (40.0) | 221,074 (37.7) |  |
| 51-60 | 221,466 (27.9) | 33,187 (25.8) | 16,225 (26.8) | 15,217 (27.2) | 166,320 (28.3) |  |
| >60 | 114,698 (14.4) | 15,851 (12.3) | 8,409 (13.9) | 6,678 (12.0) | 88,005 (15.0) |  |
| **GP experience (years)** | |  |  |  |  |  |
| 6-10 | 118,123 (14.9) | 20,015 (15.6) | 9,162 (15.1) | 8,787 (15.7) | 86,209 (14.7) |  |
| 11-20 | 346,430 (43.6) | 61,131 (47.6) | 27,436 (45.3) | 25,939 (46.4) | 250,588 (42.7) |  |
| >20 | 329,667 (41.5) | 47,376 (36.9) | 23,903 (39.5) | 21,129 (37.8) | 250,286 (42.6) |  |
| **Primary care specialist** | |  |  |  |  |  |
| No | 132,270 (16.7) | 20,773 (16.2) | 9,690 (16.0) | 9,154 (16.4) | 98,489 (16.8) |  |
| Yes | 404,080 (50.9) | 66,790 (52.0) | 31,699 (52.4) | 28,731 (51.4) | 297,058 (50.6) |  |
| Unknown | 257,870 (32.5) | 40,959 (31.9) | 19,112 (31.6) | 17,970 (32.2) | 191,536 (32.6) |  |
| **Patients seen in past hour** (quintiles) | |  |  |  |  |  |
| First hour of shift | | 129,878 (16.4) | 18,703 (14.6) | 10,952 (18.1) | 8,287 (14.8) | 97,635 (16.6) |
| 1^st^ | 202,711 (25.5) | 33,982 (26.4) | 14,569 (24.1) | 15,284 (27.4) | 149,272 (25.4) |  |
| 2^nd^ | 106,885 (13.5) | 17,973 (14.0) | 7,804 (12.9) | 7,869 (14.1) | 78,485 (13.4) |  |
| 3^rd^ | 112,784 (14.2) | 18,729 (14.6) | 8,649 (14.3) | 8,035 (14.4) | 82,773 (14.1) |  |
| 4^th^ | 161,247 (20.3) | 26,242 (20.4) | 12,184 (20.1) | 11,041 (19.8) | 119,170 (20.3) |  |
| 5^th^ | 80,715 (10.2) | 12,893 (10.0) | 6,343 (10.5) | 5,339 (9.6) | 59,748 (10.2) |  |
| **OOH shifts in past 180 days** | |  |  |  |  |  |
| First 180 days of followup | 100,772 (12.7) | 12,147 (9.5) | 9,017 (14.9) | 6,391 (11.4) | 76,796 (13.1) |  |
| 1st quintile | 159,487 (20.1) | 26,010 (20.2) | 11,379 (18.8) | 11,503 (20.6) | 118,188 (20.1) |  |
| 2nd | 136,566 (17.2) | 22,192 (17.3) | 9,969 (16.5) | 9,895 (17.7) | 100,832 (17.2) |  |
| 3rd | 124,639 (15.7) | 20,257 (15.8) | 9,298 (15.4) | 8,776 (15.7) | 92,383 (15.7) |  |
| 4th | 141,608 (17.8) | 23,707 (18.4) | 10,651 (17.6) | 10,237 (18.3) | 103,918 (17.7) |  |
| 5th quintile | 131,148 (16.5) | 24,209 (18.8) | 10,187 (16.8) | 9,053 (16.2) | 94,966 (16.2) |  |
|  | |  |  |  |  |  |
| *^1^ POCT=Point-Of-Care test; ^2^ The three columns for POCT are not independent, as one clinic consultation can include more than one POCT; ^3^ CRP=C-reactive Protein; ^4^ RADT=rapid streptococcal antigen detection test; ^5^ We used characteristics of the parents to categorise children; ^6^ GP=General practitioner; ^7^ OOH=out-of-hours.* | | | | | | |
