# Supplementary material for: Point-of-care testing and antibiotics prescribing in out-of-hours general practice: a register-based study in Denmark
Source: BMC Prim Care. 2024 Jan 23;25:31. doi: 10.1186/s12875-024-02264-0 (PMC10804570; doi:10.1186/s12875-024-02264-0)
Supplement: Supplementary file 3 — Additional file 3: Figure A1. GPs’ tendency to use POC testing, for different GP characteristics, stratified by CRP test, RADT, and urine dipsticks. Unadjusted binomial regression (relative PUT and 95% confidence interval). [file 12875_2024_2264_MOESM3_ESM.docx]

**Additional file 3.**

**Figure A1.** GPs’ tendency to use POC testing, for different GP characteristics, stratified by CRP test, RADT, and urine dipsticks. Unadjusted binomial regression (relative PUT and 95% confidence interval).

**
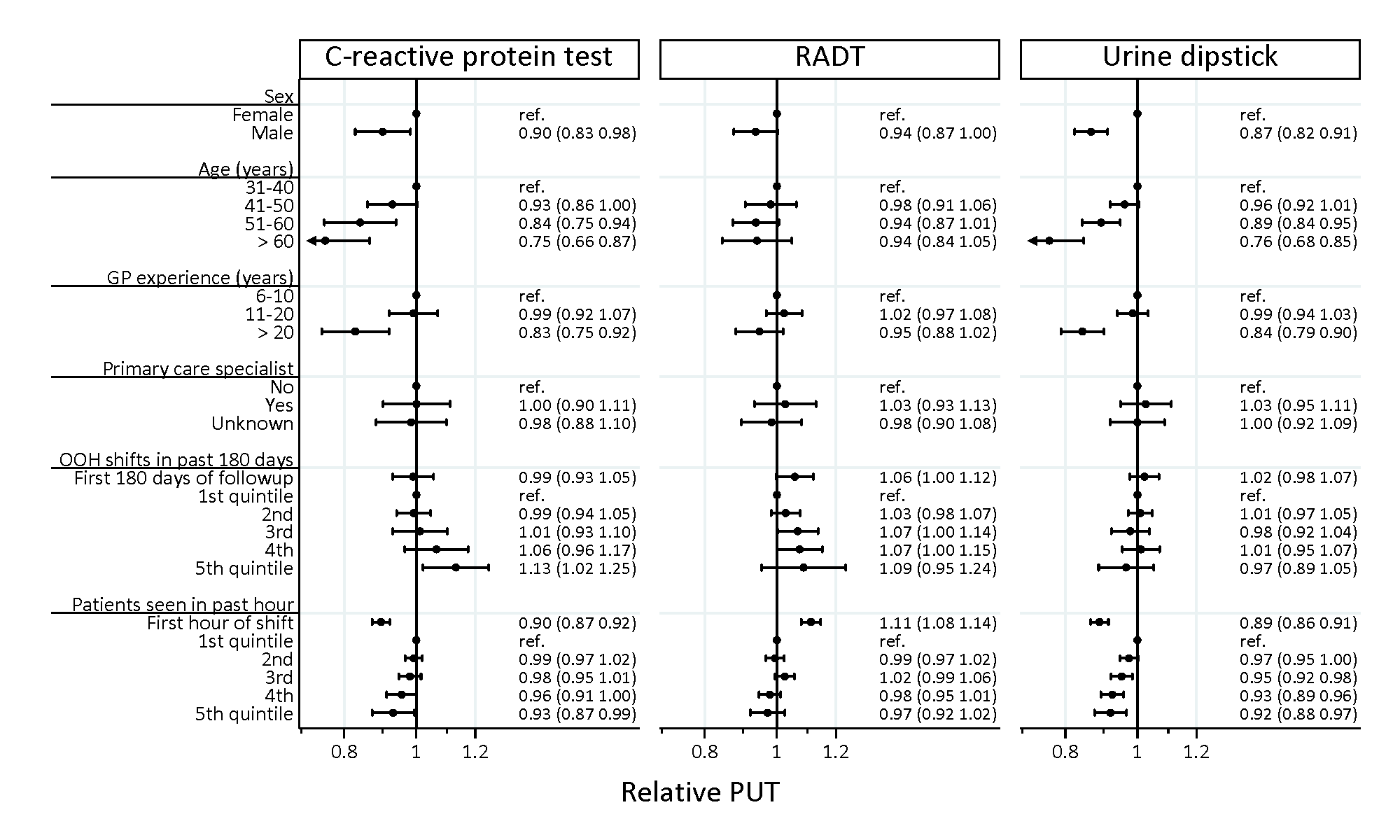
**

**PUT: Tendency to use point-of-care testing.*
