# Supplementary material for: Point-of-care testing and antibiotics prescribing in out-of-hours general practice: a register-based study in Denmark
Source: BMC Prim Care. 2024 Jan 23;25:31. doi: 10.1186/s12875-024-02264-0 (PMC10804570; doi:10.1186/s12875-024-02264-0)
Supplement: Supplementary file 4 — Additional file 4: Figure A2. Relative antibiotic prescribing rate for different levels of GP’s tendency to use POC testing (PUT), stratified by type of POC test and GP sex. Fully adjusted binomial regression (relative rate, 95% confidence interval). [file 12875_2024_2264_MOESM4_ESM.docx]

**Additional file 4.**

**Figure A2.** Relative antibiotic prescribing rate for different levels of GP’s tendency to use POC testing (PUT), stratified by type of POC test and GP sex. Fully adjusted binomial regression (relative rate, 95% confidence interval).

**
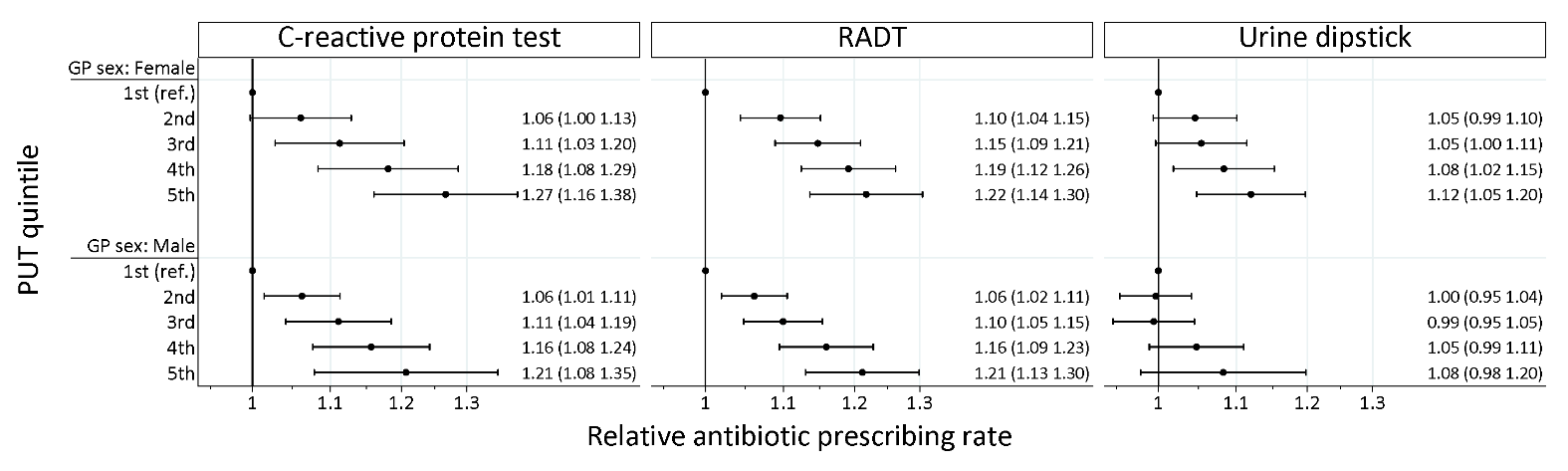
**

**PUT: Tendency to use point-of-care testing.*
